# Team‐based learning: Enhancing primary care medical education

**DOI:** 10.1111/medu.15713

**Published:** 2025-04-24

**Authors:** Dhrupadh Yerrakalva, Leila Saeed, Graham Easton, Maryam Malekigorji, Nick Fisher

## 1 WHAT PROBLEMS WERE ADDRESSED?

Despite more than 90% of clinical contacts taking place in primary care, classroom learning in medical school is often prepared and facilitated by hospital clinicians. With rising medical student numbers and over‐stretched GPs and practices struggling to offer clinical placements, our challenge was to provide students with quality campus‐based learning about real primary care problems, facilitated by GP tutors.

## 2 WHAT WAS TRIED?

Team‐based learning (TBL)^1^ offered a practical solution to maintain early exposure to authentic primary care problems, facilitated by real GPs, while maintaining clinical placements in later years. Through its flipped‐classroom approach, TBL fosters self‐directed learning and introduces students to collaborative teamwork to solve clinically relevant problems, mirroring current practice in primary care settings.

We followed Michaelson's TBL model^1^ by creating 1 hour of pre‐sessional material followed by an in‐person 10‐question test that students would sit individually and then in groups. We designed authentic and engaging application exercises, including role plays using simulated patients, unfolding cases and typical investigations correlating to the TBL topic of the day.

We developed six curriculum‐aligned topics. Over the academic year, this approach allowed us to teach 360 second‐year students over 24 sessions across 12 days.

Each in‐class TBL day included one 3‐hour session for approximately 90 students, requiring a single large flat room with tables for 8‐student teams, and facilitation by three GP tutors. Tutors were able to roam between groups in breakout tasks to facilitate discussion. This model supports small group active learning, while reducing strain on GP placements, and the need for multiple tutors across numerous practices.

## 3 WHAT LESSONS WERE LEARNED?

Student feedback showed appreciation for the collaborative team environment. Learners particularly valued clinical scenario exercises that reflected GP consultations and patient journeys. They found such real‐life application exercises within the TBL sessions beneficial, especially when they were relevant to assessment preparation such as Objective Structured Clinical Examination (OSCE).

Initially, some students were concerned that they were missing out on direct clinical experience, impacting their engagement and support for TBL. We highlighted clinical relevance by presenting scenarios from General Practice (e.g. discussing driving with a patient with suspected sleep apnoea) by practising GPs.

Some sessions in large lecture theatres posed challenges; poor acoustics and the tiered seating in rows impacted small group and tutor interaction. Smaller, flat‐seating rooms were more effective in facilitating collaboration for 90 students. In addition, some students were anxious about sharing their team's responses with the larger group, which was mitigated by offering alternative methods for asking questions anonymously (e.g. via Padlet) and praising their contributions to the wider group to build confidence.

Whereas initial TBL set‐up demanded significant faculty time to produce material and train in TBL facilitation, this investment decreased workload significantly in the subsequent year, enhancing long‐term sustainability.

Integration of digital tools including Microsoft Forms enabled tutors to identify and address student knowledge gaps in real‐time.

In summary, TBL is an effective, cost and time‐efficient alternative to practice‐based primary care teaching with authentic small group active learning in early years students.

## AUTHOR CONTRIBUTIONS

Dr Dhrupadh Yerrakalva helped in the design and facilitation of TBL sessions. He additionally helped in the drafting and editing of the paper. Dr Leila Saeed was the module lead for TBL. She helped in the design and facilitation of TBL sessions. She helped with the editing of the paper. Professor Graham Easton helped in supporting the design of sessions. He helped facilitate sessions. He helped in the editing of the paper. Dr Maryam Malekigorji helped in the facilitation of TBL sessions. She helped with the editing of the paper. Mr Nick Fisher helped in the conversion of TBL to online content. He helped in the facilitation of the sessions. He helped in the editing of the paper.

## CONFLICT OF INTEREST STATEMENT

None.

## ETHICS STATEMENT

No formal ethical approval was received. Feedback was gained to improve the quality of teaching for students. This was collected anonymously.

## Data Availability

Data available on request due to privacy/ethical restrictions. The data that support the findings of this study are available from Queen Mary University of London – Institute of Health Sciences. Restrictions apply to the availability of these data. Data are available from the authors with the permission of Queen Mary University of London – Institute of Health sciences.
